# Healthcare professionals understanding, perceptions and experiences of head and neck lymphoedema to promote self-management for patients following completion of head and neck cancer treatment: a qualitative study

**DOI:** 10.1007/s11764-025-01868-4

**Published:** 2025-07-16

**Authors:** Lauren J. Mullan, Nicole E. Blackburn, Jackie Gracey, Lynn Dunwoody, Jill Lorimer, Cherith J. Semple

**Affiliations:** 1 School of Nursing, Institute of Nursing and Health Research, Ulster University, Belfast, UK; 2 School of Health Sciences, Institute of Nursing and Health Research, Ulster University, Londonderry, UK; 3 School of Psychology, Faculty of Life and Health Sciences, Ulster University, Londonderry, UK; 4 Physiotherapy Department, Cancer Centre, Belfast Health and Social Care Trust, Belfast, UK; 5 Cancer Services, South Eastern Health and Social Care Trust, Belfast, UK

**Keywords:** Head and neck cancer, Lymphoedema, Self-management, Survivorship, Qualitative, Healthcare professionals

## Abstract

**Purpose:**

Head and neck lymphoedema (HNL) is a chronic and often debilitating consequence of head and neck cancer (HNC) treatment. Despite the chronicity, it is often under-recognised and under-treated. There is a finite resource of specialists to provide HNL intervention, necessitating self-management as an important pillar of patient management. This study aims to explore healthcare professionals (HCPs) understanding, perceptions and experiences of HNL management to promote self-management and adherence to HNL interventions in patients following completion of HNC treatment.

**Methods:**

Eight focus groups were conducted with HCPs (*n* = 39) from different professional disciplines, acute and community settings and countries. Reflexive thematic analysis was adhered to in the development of key themes using an inductive approach.

**Results:**

Two main themes were established: (1) ‘Raising awareness of HNL as a chronic and complex condition’ and (2) ‘What are the solutions for promoting HNL self-management?’ Data demonstrated that HCPs lack confidence and understanding in providing HNL support. There was a lack of clarity towards roles and responsibilities of HNL management among HCPs, with many having a desire to be better equipped to promote patient self-management and adherence.

**Conclusion:**

HCPs involved in care delivery of patients with HNC require education and training on HNL, to support patient and family engagement with HNL self-management. To advance adherence and compliance of HNL self-management, there is a need to develop a dedicated HNL self-management intervention, integrating theory and evidence.

**Supplementary Information:**

The online version contains supplementary material available at https://doi.org/10.1007/s11764-025-01868-4.

## Introduction

Treatment for head and neck cancer (HNC) can incorporate multiple modalities including surgery and or radiotherapy, chemotherapy and immunotherapy [1, 2]. The affects of these treatments can be detrimental, impacting functional and psychosocial well-being [3]. Head and neck lymphoedema (HNL) is a common and often debilitating consequence of HNC treatment, effecting up to 90% of patient’s post-treatment [4, 5].

Despite this prevalence, HNL is often under-recognised and under-treated, posing a critical issue to address for this cancer population [5, 6]. HNL can be described as an accumulation of protein rich fluid within the interstitial spaces of the head, face and neck [7–9]. The condition can develop externally, internally or in combination [10]. External HNL relates to the soft tissues of the face and neck, whereas internal HNL presents within the oral cavity, pharynx and larynx [8, 11]. Symptoms of HNL commonly include tightness of the skin and, reduced mobility of the head, neck and shoulders [12, 13]. Furthermore, HNL is associated with difficulties in speech, swallowing, changes in appearance and breathing challenges [5, 7]. A recent systematic review highlighted such difficulties can lead to negative impacts on patients’ overall well-being and health-related quality of life (HRQOL) [14].

Although there is a growing interest in the field of HNL to develop effective and efficient self-management interventions for patients, HNL management is still very much in its infancy [4, 8]. Research indicates there are global variations in the standard of care for HNL management [1], but the current perceived model of best care employed in many international settings is complete decongestive therapy (CDT) [7, 15, 16]. Furthermore, internationally there is no standard pathway for HNL management or referral processes, therefore patients are often only referred for treatment when HNL symptoms are severe [1, 17–19]. There is also limited availability of HNL specialists and lack of funding, making them a finite resource, resulting in patients waiting longer for appointments [14, 20]. The current body of literature identifies that although there are global differences surrounding HNL and its management, it is recognised that HNL specialist therapists generally consist of HCPs with post-graduate training in lymphoedema management and directly involved in providing support and treatment to patients with HNL, including lymphoedema physiotherapists, speech and language therapists, occupational therapist and/or nurses [10].

HNC has been highlighted as the 6th most prevalent cancer globally, with 12,759 cancer cases reported each year in the United Kingdom (UK) [14]. The growing incidence rates of HNC and progressive treatment advancements evidence that people are living longer with the associated treatment-related side effects [21, 22]. The chronicity and complexity of HNL means this condition requires ongoing management [8, 23]. Moreover, the unique burden of HNL, combined with the extensive number of appointments that HNC patients are required to attend throughout the treatment pathway, highlights the important role of self-management [7, 24]. Of note, HCPs approaches to managing chronic conditions have shifted, moving from traditionally professional led to collaborating with patients and family, encouraging them to take an active role through self-management and individualised care [16, 25–27]. The relationships between patients and their family members are emphasised as being fundamental in competently managing chronic conditions, depicted by the Individual and Family Self-Management Theory [27, 28]. Family members can play an influential role in providing emotional support and motivating through positive reassurance [27, 29]. Furthermore, HCPs alongside family and peer support have an instrumental role in patients’ individual ability to manage their chronic condition, such as HNL [23, 28, 30, 31].

Despite self-management having positive effects on HRQOL and functional outcomes for numerous chronic conditions [25, 26, 31], there are limited effective evidence-based self-management interventions for HNL [14, 25, 31], to include how best to embed HNL self-management into routine clinical care [14, 23, 28, 30]. Adherence to existing HNL self-management strategies has been reported as poor, particularly within home-based settings which consequently can increase the burden placed on HCPs with increased hospital admissions relating to HNL [14, 20]. Evidently, there is a need to acquire an in-depth understanding of what enables long-term behaviour change in patients with HNL to promote adherence and motivation of HNL self-management strategies [14, 31, 32].

Behavioural change interventions have been identified as being more effective when tailored and personalised to the individual patient’s needs, implying that patients should not be solely responsible for successful HNL self-management [7, 28]. Consequently, HCPs have been identified as having an influential role in promoting self-management of HNL; therefore, it is essential to consider how HCPs perceive self-management of HNL [7, 33, 34]. Studies focussing on self-management within general chronic conditions have identified that although HCPs may view their role within self-management as something positive, they may lack the necessary skills, training and resources to effectively support self-management of chronic conditions [28, 34]. This clearly emphasises the importance of including HCPs experiences, perceptions and understanding of HNL management.

This study aimed to explore HCPs’ understanding, perceptions and experiences of HNL management to promote patients’ self-management and adherence to HNL interventions. The objectives were to:

- Explore HCPs’ experiences of providing HNL management within routine clinical practice for patients following HNC treatment.
- Explore HCPs’ perspectives of key barriers and facilitators in promoting HNL self-management for patients following HNC treatment.
- Identify HCPs’ perceptions of how patients following treatment for HNC can be best supported to promote engagement and adherence to HNL self-management.

## Methods

This study utilised focus groups, which were conducted between June and December 2024 with a range of HCPs. This permitted exploration, discussion and sharing of expertise in HNL management [35] and clinical experiences [36]. The study followed the Consolidated Criteria for Reporting Qualitative Research (COREQ) guidelines [37].

### Selection of participants

Using purposive sample techniques, a range of HCPs working across different professional disciplines, acute and community settings and different countries were recruited. Purposive sampling included participants who were all directly involved in providing care to patients who had previously completed treatment for HNC. Participants included specialist HNL therapists such as lymphoedema physiotherapists and speech and language therapists (SLT), and non-specialist HNL therapists including oncology consultants, oral and maxillofacial surgeons, restorative dentists, nurses, dieticians, and radiographers. Participants were considered specialists in HNL management if they delivered HNL interventions as part of routine practice and had specific post-graduate training in HNL management (see Table 2). Participant eligibility is detailed in Table 1. Local collaborators from the UK were from participating health and social care trusts consisting of one HNC Clinical Nurse Specialist (CNS), and two advanced clinical specialist lymphoedema physiotherapists in HNL. To maximise and garner international participation, enhancing diversity various recruitment channels were employed, including targeted social media advertisement, and participant information packs sent by local collaborators to HCPs within their professional networks. A suitable date and time were arranged for each focus group by the lead author (LM), and written consent was obtained for all participants.

**Table 1:** Study inclusion and exclusion criteria

Inclusion criteria	Exclusion criteria
• Registered HCP • Directly involved with providing care to HNC patients within hospital or community settings • Ability to provide informed consent	• No clinical experience in HNC management • No valid healthcare registration • Healthcare placement student

### Data collection

The lead author (LM) developed a topic guide which was informed by extant literature and further refined by the expertise of the research team including clinicians and experienced researchers (CJS, NEB, LD, JL). Patient and public involvement (PPI) refers to active participation of patients, career and members of the public in research to ensure patients expectations, need and preferences are met, which is positively promoted to improve the validity and relevance of research [3, 35]. Within this study, PPI was integral with the topic guide being reviewed by two HNC patients and two HCPs to corroborate content and meaning of the topic guide (Online resource 1). Key perceptions from PPI facilitated refining introductory questions to focus on HNL and additional questions to garner understanding of internal HNL. Focus groups (*n* = 8) were conducted online (*n* = 7) and face-to-face (*n* = 1) by three of the research team (LM, CJS, NEB). All focus groups were organised in relation to size, accommodating a maximum of eight participants to optimise engagement and contribution during data collection. Where possible focus groups were organised with diversity across specialist and non-specialist, being achieved in six out of the eight focus groups. Participant demographic information was collected at the beginning of each focus group (Table 2). The first author (LM), a registered therapeutic radiographer and PhD researcher, was not known to the participants and completed qualitative training prior to the commencement of the study. Other members of the research team (CJS & NEB) have extensive experience in qualitative research to include focus groups. Focus groups were completed when no new key information, codes or themes were emerging from the data, in keeping with the values and assumptions of reflective thematic analysis [38]. The first author (LM) made reflective notes after each focus groups to become familiar with the data, promote rigour and ensure all topics were explored.

**Table 2:** Sample characteristics of the health and social care professionals

HCP ID	Gender	Professional discipline	Years qualified (years)	Years working directly with HNC (years)	Dedicated post-graduate training in HNL
1	Female	Radiographer	11–15	0–5	No
2	Female	CNS	11–15	0–5	No
3	Female	CNS	11–15	0–5	No
4	Female	Consultant radiographer	16–20	6–10	No
5	Female	CNS	21 +	21 +	No
6	Female	SLT	6–10	0–5	No
7	Female	SLT	21 +	21 +	No
8	Female	CNS	21 +	6–10	No
9	Female	CNS	21 +	6–10	No
10	Female	CNS	21 +	6–10	No
11	Female	Radiographer	11–15	6–10	No
12	Female	CNS	21 +	11–15	No
13	Female	Dietician	0–5	0–5	No
14 15 16 17 18 19 20 21 22 23 24 25 26 27 28 29 30 31 32 33 34 35 36 37 38 39	Female Female Female Female Female Female Female Female Female Female Female Female Male Male Male Male Female Female Female Female Female Male Female Female Female Female	Specialist physiotherapist Specialist physiotherapist Specialist physiotherapist Specialist physiotherapist Nurse SLT Specialist physiotherapist Specialist physiotherapist Specialist physiotherapist SLT Nurse SLT Dentistry Oral and Maxillofacial surgeon Dentistry Consultant oncologist Oral and Maxillofacial surgeon Dentistry Dentistry Oral and Maxillofacial surgeon Oral and Maxillofacial surgeon Faculty member Physiotherapist Physiotherapist Physiotherapist Physiotherapist Physiotherapist	21 + 21 + 21 + 16–20 16–20 21 + 21 + 21 + 11–15 21 + 21 + 11–15 21 + 21 + 0–5 16–20 6–10 11–15 16–20 21 + 21 + 21 + 16–20 11–15 11–15 6–10	21 + 6–10 6–10 0–5 6–10 21 + 11–15 11–15 6–10 6–10 21 + 11–16 0–5 6–10 0–5 11–15 0–5 0–5 0–5 6–10 21 + 16–20 6–10 0–5 0–5 0–5	No Yes Yes Yes No Yes Yes Yes Yes Yes Yes Yes No No No No No No No No No Yes Yes Yes Yes Yes

Findings are discussed under two main themes: (1) raising awareness of HNL as a chronic and complex condition and (2) what are the solutions for promoting HNL self-management?

*CNS* Clinical nurse specialist, *SLT* Speech and language therapists

### Data analysis

Audio recordings were transcribed verbatim by either first author (LM) or an external expert transcriber. Transcripts were checked for accuracy (LM) and subsequently imported to NVIVO 15 [39] with any identifiable information removed. To promote rigour all transcripts were read independently by either CJS or NEB [40]. Data analysis for this study adhered to Braun and Clark’s [40] six step approach to reflexive thematic analysis. The written reflective notes made after each focus group were expanded upon when reading the transcripts to ensure rigour and reliability [41]. The first author (LM) conducted initial coding using NVIVO 15 [39], with categories and initial themes being discussed and refined with the research team to ensure that they aligned with the study’s overall aim and objectives (CJS, NEB, JL). All themes identified were iteratively discussed and refined through critical dialogue to provide accurate representation and interpretation of participants. This process followed the COREQ guidelines, ensuring transparency and rigour [37].

### Ethical approval

All potential participants were provided with written information packs, which included study invite, participant information sheet and consent form. Participants were made aware of their right to pause, reschedule or cease the interview. A distress protocol was developed; however, this was not required. Two researchers were present to enable focus groups to be moderated by one of the researchers, promoting engagement with all participants, managing group dynamics to minimise dominant voices overshading others, with conversations guided to yield interactive discussions [36]. Ethical approval was received from the Office for Research Ethics Committees Northern Ireland (OREC: 23/NI/0096).

## Results

A range of HCPs were recruited (*n* = 39), consisting of 34 women and 5 men, with global representation from United States of America (USA) (*n* = 6), Sweden (*n* = 1), United Kingdom (UK) (n = 31) and Australia (*n* = 1). The sample included specialist physiotherapists (*n* = 12), radiographers (*n* = 3), speech and language therapists (SLT) (*n* = 5), clinical nurse specialists (CNS) (*n* = 7), oral and maxillofacial surgeons (*n* = 4), consultant oncologists (*n* = 1), restorative dentists (*n* = 4), oncology nurses (*n* = 2) and dieticians (*n* = 1). Participant characteristics are presented in Table 2. On average focus groups lasted 40.21 min, ranging between 27.25 and 71.57 min.

### Theme 1: Raising awareness of HNL as a chronic and complex condition

Participants highlighted the importance of raising the awareness of both the chronicity and complex consequences of HNL. This theme is discussed under two sub-themes: (1) ‘lack of awareness and knowledge among non-specialist HCPs’ and (2) ‘importance of HCPs raising awareness for patients and family’.

### Subtheme 1.1: Lack of awareness and knowledge among non-specialist HCPs

Most participants recognised that non-HNL specialists, working in HNC, had low levels of confidence to provide information and support to patients and family members regarding HNL and its management. Subsequently, non-HNL specialist often did not perceive it was within their remit to support and educate patients regarding HNL. Specialist HNL therapists perceived this lack of information provision to patients by HNC team members created frustration in patients’ acceptance surrounding HNL management, often resulting in unrealistic patient expectations. Furthermore, some professionals highlighted that on occasions patients were being provided with inappropriate treatment, for example, prescribed antibiotics instead of onward referral for HNL management.

I had a classic example last week, he [a patient] was a few months post-treatment and he had very classic lymphoedema [..], rather than come straight to us, he had been seeing his General Practitioner in the community since May, and every month since May his GP has been giving him antibiotics for his infection in his neck. He [the patient] thought well, the antibiotics aren’t working so I think it’s cancer and he came and saw me. I couldn’t tell you how classic this is, got the doctor to double check and then had to send a fairly terse email to his GP, but it’s a common thing that I am dealing with, yeah [HP21, SLT].

Specialist HNL therapists demonstrated an enhanced understanding of the biopsychosocial challenges associated with HNL including functional impairments, changes in appearance, negative impacts on emotional well-being and overall HRQOL. Nonetheless, even among HCPs with specialised training in HNL, distinct geographical differences existed in HNL knowledge and awareness. Within the USA, HNL services were predominantly delivered by SLTs, whereas across the UK, delivery models were mainly physiotherapist-led. More often, professionals from the USA seemed to have a greater accessibility to specific HNL training, and demonstrated a more in-depth understanding and knowledge, especially of internal HNL in contrast to most other professionals within the study. Additionally, our participant from Australia who was a SLT, also had insight into HNL management. Overall, non-HNL specialists identified a poor understanding of internal HNL, highlighting their main focus when referring patients for HNL treatment was based on external symptoms.

We have had a few patients who were having difficulties with their speech and swallow, and they evidently had lymphoedema on the outside. We did put a referral in to say we also felt there may be something being affected on the inside as well. But we don’t really have that knowledge. I suppose I would just always go on what I am seeing externally and that would kind of be the trigger for me in referring for lymphoedema. Internal HNL isn’t really on the radar for us. That maybe would be more consultant led or even monitoring from a GP who could observe it [HP11, CNS].

Furthermore, most HCPs identified a lack of clarity regarding the role of HCPs within HNL management and no clearly advocated assessment tools or referral pathways. Non-HNL specialists reported a lack of knowledge and awareness on how best to provide HNL support to patients or family members. Nonetheless, most participants reported that the role of the CNS was predominately responsible for the provision of basic patient information on HNL, screening for HNL and onward referral to specialist therapists for HNL intervention. This was in contrast to the role of specialist HNL therapists, who were perceived as conducting a detailed HNL assessment, delivery of tailored intervention to incorporate elements of CDT, patient education and promotion of skills training required for HNL self-management. With the exception of the CNSs and specialist HNL therapists, most participants reported a lack of clarity in the role other disciplines within the HNC team could play in providing support and promoting HNL self-management. This included SLTs (not specialised in HNL management), dieticians and radiographers. Additionally, medical HCPs stated that they did not perceive HNL management to be their responsibility, defaulting this responsibility to CNS who they perceived to be better informed to provide patient HNL support.

This is something than can act as a barrier and may be something we need to bring into the education side but perhaps the clinicians don’t always really know about it [..] but they don’t always know about HNL and they very much undersell it to patients and they will often put in letters ‘oh lymphoedema’ but they don’t think its their role to a thing about it. They don’t tell the patient how to manage it, they sometimes don’t even refer them. They just acknowledge the fact that the patient has it [HP5, Radiographer].

### Subtheme 1.2: HCPs raising awareness for patients and family

The majority of HNL specialists recognised the importance of raising the awareness and knowledge of HNL for patients and family members, to include the cause, complexity and chronicity. Specialist HNL therapists also identified a lack of patient awareness of why they were referred to a HNL specialist, hence no insight into their role within HNL self-management.

Patients have no idea. Patients come in expecting to be physically drained with a needle. They have no idea what or why they are coming in for. A lot come in going I don’t even know why I am here, I’m coming to physio. They are expecting again a more traditional side of things, they have no idea why they are here. But they need to know they have an important role in the management of it and how they interact with what we give them and if they continue on with it. That makes a big difference [HP 15, Physiotherapist].

HCPs explained that patients and family members often associate post-treatment swelling as cancer recurrence, especially swelling which occurred in the submental region and coincided with an increase in symptom burden (pain, tightness of skin and restricted range of motion). Consequently, some participants reported the importance of providing timely patient education of HNL to reduce anxiety and distress. Specialist HNL therapists highlighted that information should also relate to the chronicity of HNL, to reduce frustration of yet another burden to overcome when it presents after completion of HNC treatment.

So they instantly assume, obviously its submental, em as soon as they get that and it starts to happen they panic and think that they have a em a cancer recurrence or progression and em that’s a big challenge to overcome, just like kind of reassuring the patient [HP 4, CNS].

Although education was highlighted as fundamental to promote HNL adherence, there was no clear consensus on the best time point to deliver this education. Most HCPs emphasised that introducing HNL education at multiple points along the treatment pathway, in small ‘bitesize’ pieces, could facilitate patient recall and understanding therefore help to promote HNL self-management. Alongside this, a few HCPs did state that there was value of patients receiving HNL education early to provide clear expectations. However, many professionals did express concerns that receiving information ‘too early’ may result in lack of recall due to the overwhelming amount of information and differing patient priorities when newly diagnosed. HCPs did feel that the timing of HNL education was not a ‘one size fits all’ approach, with the need to assess patients’ emotional readiness to receive the information, further indicating that HNL education needs an individualised approach.

I think the best time would be early and then ongoing because they’re inundated with so much information early on, I question how much of it really sticks because I’ve had patients come from the same office and one of them, sometimes they would say “Nobody ever told me anything about this” and other people would say “Oh yeah, they warned me about this” and I doubt it’s the office themselves educating differently, it’s just what sticks with the patient in the moment, so early and then ongoing a needed would be best, I think [HP 39, Physiotherapist].

### Theme 2: What are the solutions for promoting HNL self-management?

Participants identified the importance of the provision of education for patients, family members and non-specialist HCPs to promote HNL self-management. This theme is discussed under three subthemes: (1) ‘strategies to promote HNL self-management’, (2) ‘consistency of information and practice is key in promoting HNL self-management’ and (3) “delivery mode of information to promote HNL self-management’.

### Subtheme 2.1: Strategies to promote HNL self-management

Most participants explained the importance of educating patients on the important role they have within HNL self-management. Thus, providing patients with a sense of control, therefore empowering them to adhere to HNL self-management techniques. Some HCPs commented further on the importance of understanding their patients’ learning preferences and factors that promoted engagement. This could entail preferred mode of information delivery, personal motivation factors and ability to incorporate with daily routine to promote adherence with HNL self-management. Some specialist HNL professionals highlighted the importance of ensuring information was presented to patients and family in ‘bite-size’ pieces as to not overwhelm whilst also using appropriate terminology. HCPs recognised the importance of taking into consideration patients health literacy.

Knowing they can have a role in it, is helpful knowing that there is an ability to control this. That they have a role in treating this condition and it is so important. That can help because they will have had no control up to now. This is something they can impact on. Sometimes if it is clear to them I find they take something positive from that. So a lot of patients can respond better I think to self-management because actually they are pleased to be able to do something about it [HP 14 Physiotherapist].

Patient compliance with HNL self-management was also reported as variable, incomplete and often considered as ‘poor’ by many HCPs. Participants perceived that HNL self-management compliance and adherence can be dependent not only on the factual information provided by professionals, but on the delivery style and skill-set of the professional to engage in person-centred care. Furthermore, adherence was perceived by HCPs to be dependent on the patient’s individual motivation and attitudes towards HNL. Strategies employed by specialist HCPs to enhance patients’ motivation included emphasising benefits of self-management, using objective measurements to demonstrate improvements and employing peer support to empower patients to adhere to self-management strategies,

And as always the compliance with HNL will vary amongst different patients em, and some will engage more and I suppose that is when the education has to come in hand in hand to encourage them to engage with their self-management [HP3, CNS].

Most HCPs recognised that family members could provide a positive support system in encouraging and promoting patients to ‘keep going’ and be persistent in their self-management strategies. Specialist HCPs further highlighted that by ensuring family members were informed and understood the positive benefits of self-management on HNL symptoms, this could promote engagement and adherence. While many HCPs reported the role of family members as predominately providing emotional support in HNL, a few professionals did suggest that family members could practically support patients with HNL self-management when patients experienced dexterity issues. Practical support included physically massaging the affected area and help with positioning of compression garments. Of note, a minority stated that self-management should be solely dependent on the patient, suggesting that by engaging family members this could create a co-dependency.

If they have a partner, or somebody coming in with them and if you can get them on board and they have the understanding of the importance of it. [..] If you can get family on board too then they are there to encourage them or even do some of it because we often find family members are keen to do something because they are very much on the sidelines. So sometimes you can involve them in doing a little bit of the massage and things. Or even encourage them or just being the prompt of have you done your exercises, have you done your massage, right lets get on with it. So you can definitely use family too [HP13, Physiotherapist].

The majority of specialist HNL therapists were cognisant that objective and subjective HNL improvements motivated patients to engage and adhere with self-management strategies. This included patients seeing visual improvements with reduced swelling, clothing feeling looser around the neck and positive changes in objective measurements taken by HCPs. A few specialist professionals stated that although patients were often motivated through improvements of their symptoms, some patients could become fixated on the use of objective measurements. Consequently, hindering their adherence and motivation with HNL self-management if improvements did not meet their expectations or there were no improvements at an appointment.

We take photographs in our clinic so we can actually show them the improvements and it is visible. So this is actually something that it promotes self-management as it’s ok I’m doing something right. But also even if photographs are not significantly different they sometimes rely on feeling so ok it feels better because there is more movement there less tightness or puffiness [HP19, SLT].

### Subtheme 2.2: Consistency of information and practice is key in promoting HNL self-management

Most participants stated that providing patient information with a consistent message on causality and management throughout the cancer trajectory was important to promote HNL self-management, mitigating confusion and promoting adherence to self-management. There was an overwhelming consensus from HCPs of the lack of, and desire for high-quality evidence-based HNL resources for patients to promote self-management. In addition, some HCPs specialising in lymphoedema management highlighted the importance of educating all HCPs involved in HNC care on what HNL is and the recommended practices to ensure that patients are receiving consistent high-quality evidence-based information and self-management instructions.

We need to better educate all the providers on what lymphoedema is and what the recommended practice is because I do think when patients hear a consistent message they’re better off. So I think if a patient is seeing their oncologist and they are told this is what is recommended and they go then to see a lymphoedema provider having that information the same [..] and we have all mentioned that we treat lymphoedema the same way is important. However I do wonder at the minute about the consistency of interventions provided for lymphoedema. A person might go to see a lymphoedema therapist and the treatment recommended is not necessary the standard care then this is the challenge [HP 36, Physiotherapist].

As reported by specialist HNL therapists, a potential reason for lack of consistent information provided at a patient-level is likely due to global variations, with no clear consensus towards evidence-based practice for HNL. This was clearly depicted in the data, as some specialist HCPs stated that initially they would introduce all elements of CDT; however, CDT would soon be tailored, as different elements suited different individual patients. A few HNL specialists indicated they also incorporated taping and manual lymphatic drainage as their main treatment for patients. In contrast, others reported that exercises for the head, neck and shoulders was the most common intervention element used, with only a few reporting on compression devices being incorporated into the treatment plan. Most HCPs indicated that patients found compression devices uncomfortable and difficult to tolerate, resulting in inconsistency of use with patients. Despite this, these devices were still introduced to patients as part of the complete CDT intervention.

I’ve seen a lot of variability in that I’ve had providers that actually provide only brief instruction on techniques. I’ve also had providers tell patients to do specific massage like techniques or even tell patients to get a compression garment so I definitely do see great variability in current practices which is challenging [HP 35, Physiotherapist].

### Subtheme 2.3: Delivery mode of information to promote HNL self-management

Tailored education to promote HNL self-management was reported as fundamental by most HCPs. Participants outlined that presenting personalised HNL information would aid normalising of this treatment-related side effect and motivate patients to adhere to HNL self-management.

I think a lot of it is driven by the patient because everybody learns a little differently. I think face to face so that if they have questions is very convenient but again, they’re being educated about a lot of different things at the same time, so, having some sort of take away brochure, a handout, something they could kind of digest on their own later and then come back again if they have more questions is helpful [HP 36, Physiotherapist].

HCPs highlighted, that to actualise delivery of personalised information and aligning this to patients preferred learning styles would mean having a range of patient resources for HNL self-management. Useful modes of intervention delivery identified by professionals were dedicated HNL written leaflets, diagrams, online resources, videos, face-to-face appointments with a specialist therapist, and peer support. Some HCPs suggested the benefit of a tailored HNL patient resource, presented as a one-page infographic including basic information on HNL and its management, with a QR code enabling patients to access further detailed information and a video. Moreover, HCPs highlighted the importance of using simple and clear information, avoiding medical jargon. A few HCPs highlighted the utility of analogies to avoid using medical terminology such as plumbing systems and traffic lights to normalise HNL and minimise confusion with HNL education. This was in an effort to ensure patients were able to understand the information, promote acceptance of the condition and facilitate adherence to self-management.

I’ll describe it, the lymphatic system as your body’s plumbing and drainage system to get rid of waste and debris, as I call it. But also I tend to give an analogy such as you know, a motorway is blocked because there’s roadworks or there’s been an accident and so, the traffic has to divert through alternative A roads, and I kind of give that analogy that at the moment everything’s blocked in your normal plumbing and drainage system so we have to divert the fluid over time physically ourselves by moving that fluid from an area that doesn’t work to an area that does work and eventually, the body will recognise that new route itself but it needs to have constant repetition to begin with until the body starts to recognise that new pathway [HP 25, SLT].

## Discussion

This novel study is the first to the authors knowledge to identify HCPs understanding, perspectives and experiences of HNL self-management. The findings emphasise that more often HNC team members lack confidence and understanding of HNL, especially internal HNL, with the exception of some specialist HNL therapists. However, professionals have a desire to be better equipped with evidence-based information and tools to provide to patients throughout the HNC treatment pathway to promote HNL self-management, which are currently lacking. HNL self-management is further hindered by a lack of both embedded pathways in routine care and clarity on the roles and responsibilities across HNC team members. The findings within this study identify the important role of family members in providing mainly emotional support and motivation to promote adherence to HNL self-management techniques. To facilitate HNL adherence, there is a need for accessible information and skills training for patients, tailored to preferred learning style, available at multiple timepoints, especially early post-treatment when HNL is most likely to present and manageable within their daily schedule.

This study identifies a lack of clarity among HCPs concerning the role and responsibility of HNL management. Global differences existed regarding the role of HCPs in HNL management, with SLTs playing an integral role within the USA healthcare system, in contrast to across the UK, where the main responsibility resides with specialist physiotherapists for the management of HNL and CNS for referral. This is consistent with the broader literature highlighting that globally there is no single modality treatment, referral process or standardised clinical pathway for HNL management [1, 4]. Nonetheless, it is portrayed by specialist therapists that all HCPs involved in HNC care have a role to play in the management of HNL. Early intervention has been identified as positively impacting patients’ symptoms and ability to self-manage HNL [10, 42]. In the absence of clarity regarding HCPs role, there can be potentially negative consequences on patients’ accessing quality information, timely access to HNL services to include training on self-management, leading to additional treatment related symptom burden and overall poorer HRQOL [7]. Consequently, HNL may not be identified until symptoms are more severe and potentially irreversible [7, 10, 42].

All professionals involved with HNC patients along the treatment pathway need to be aware of HNL and proactively identify potential signs and symptoms to action referrals early, consequently minimising symptom burden for patients and sharing the responsibility in the management of HNL [7, 43]. This presents a challenge, as many non-lymphoedema specialists both within the acute and community HNC team did not deem it within their scope of practice and lacked confidence to provide education and support for HNL management. This may in part account for the lack of patients being referred for HNL management, despite the propensity of this debilitating post-treatment effect [22, 44, 45]. Other obstacles to early equable referrals for HNL include fragmented pathways and need for comprehensive assessments of HNL, taking account of the physical, functional and socioemotional impacts of HNL [22, 46]. Furthermore, HCPs lack of knowledge, training opportunities and evidenced-based accessible patient resources on HNL mitigate for lack of HNL self-management opportunities. This can create a chasm, resulting in HNL symptoms being more severe and, or anxiety for the patient with fear and misunderstanding that their cancer had returned, emphasising the importance of clear and early education from HCPs regarding HNL [4, 22, 42]. Consideration of the different international healthcare systems and structures must be addressed in the development of a HNL intervention. Providing recommendations for clinical practice based on evidence-based research can potentially help mitigate the global lack of standardisation within HNL care [1, 6, 46]. These recommendations could be employed to ensure standardised HNL resources and care at country and trust level.

Moreover, in this study, it was evident that most HNL referrals were made because of external HNL. This is disconcerting as previous research indicated that external HNL accounted for 2% of HNL cases, 35% internal HNL and 60 + % associated with a combination of both internal and external HNL [8]. Educating HCPs on HNL is therefore vital to increase their knowledge and understanding of both internal and external HNL. Lack of knowledge and confidence within HCPs in cancer care is a critical issue because patients require open, honest and tailored education with HCPs [43]. Current literature acknowledges that person-centred communication between HCP and patient is necessary for quality care and promoting self-management [43, 44]. Clinical experience alone cannot establish effective delivery of person-centred cancer care [43, 44]. There is evidently a need to provide HNL evidence-based training across the multidisciplinary team to effectively raise awareness of both external and internal HNL and promote equal opportunities in accessing intervention to promote greater compliance with evidence-based HNL self-management [44, 45].

HCPs acknowledge the lack of, and need for a patient-focused intervention to promote HNL self-management. To advance the effective and efficient delivery of complex interventions in contemporary healthcare settings, an intervention development framework should be utilised [46, 47]. To promote acceptance and compliance of healthcare interventions, it is essential that the complexity of the healthcare structure, role and function of the HCP, patient and family members role within healthcare delivery are careful considered when developing new interventions [47]. These complex intervention development frameworks [48, 49] ensure patient and public involvement is central, which is especially important for this population with HNC patients predominately presiding from lower socioeconomic backgrounds, associated with poorer health literacy [50]. Health literacy is strongly correlated to poor health and HRQOL; therefore, when developing HNL resources, they should be accessible, understandable, culturally appropriate, use clear language and visual aids [51, 52]. These are vital considerations in promoting compliance with self-management in this cancer population [50, 51]. To promote relevance of HNL resources, patient and public involvement is integral, alongside evidence and theory integration [52, 53].

Within the HNC population self-efficacy can be low, emphasising the importance of positive affirmation and empowerment to encourage engagement and adherence [25, 50]. Previous studies within chronic conditions have identified self-efficacy as a vital psychological factor in facilitating patients to navigate self-management of their symptoms and integration into daily routine [26, 27]. By taking strides to increase patient’s self-efficacy, HCPs can encourage patients to believe in their ability to manage their symptoms, providing motivation and behaviour change [18, 22]. Furthermore, an element of effective integration of self-management into daily life and increasing self-efficacy is including techniques of trial and error and goal setting [25, 27, 32]. Subsequently, patients with HNL may benefit from the integration of individualised behaviour goal setting, improving their adherence to specific self-management strategies and therefore promoting positive long-term behaviour change. When patients have a sense of ownership in their management of HNL, combined with increased knowledge, this can contribute to better clinical outcomes and reduce requirement for additional advanced lymphoedema specialised care [45].

Previous studies based on chronic conditions support the involvement of family members to facilitate self-management, as the involvement family can improve self-efficacy and promote compliance [25, 50]. Consequently, this study identified the important and influential role that family members can have in the management of HNL, by providing emotional support and motivating through positive reassurance. The role of family members in HNL management should be encouraged and promoted by HCPs, as greater levels of family support can improve compliance and control over self-management strategies for patients with HNC [3, 26].

### Clinical implications

Self-management has been described as having an imperative role given the chronicity and lack of specialist HNL professionals [50, 52–54]. Considering previous reporting of poor or partial adherence to HNL home-based interventions, the perspectives and role of HCPs directly involved in the care of HNC patients is vital in the planning and developing of a tailored HNL self-management intervention. Self-management resources, should consider health literacy, be available in a range of delivery modes to accommodate patient learning styles, and provide ‘bite sizes’ of information. HNL resources should be accessible at multiple timepoints but especially early post-treatment, to not only enhance engagement but also promote adherence and reduce distress with the common misunderstanding between HNL symptoms and disease recurrence [6, 54, 55]. It is important that family members are abreast of this tailored information to enable provision of emotional support and motivation to help mitigate the impact of HNL [7, 43].

Despite the evident need for adequate patient education regarding HNL to promote self-management, HCPs often lacked awareness surrounding HNL causality and management, how to comprehensively assess patients and where to refer patients for HNL management. This study provides the HCP perspective, identifying a need for multidisciplinary training opportunities to promote confidence and awareness of HNL and its management among non-specialist HCPs. Additionally, there is a need for embedded referral pathways within the healthcare systems. This study demonstrates the need for a dedicated HNL resource to raise HCPs confidence in providing self-management support to patients and raising awareness of what patients can do whilst waiting for specialist HNL therapist appointments. Ultimately, reducing the burden placed on the finite specialist HNL workforce.

### Study strengths and limitations

This study addresses an important gap in the literature with the lack of research exploring HCPs knowledge, attitudes and treatment practices in the management of HNL. The study findings are reflective of HCPs experiences of HNL management across various professional disciplines, with international representation. While most participants in the study were from the UK, the global representation provided diversity and richness of HCPs experiences and perspectives of HNL management from a range of countries, incorporating potential cultural differences to consider. Although focused on HNL, HNC treatment is associated with a vast array of treatment-related side-effects [7, 22]. The principles to promote self-management from this study may also be applicable to future research on additional HNC treatment-related effects such as eating challenges, speech, swallowing and breathing difficulties [2, 3]. As with most studies there are also limitations to consider. Participants from USA were professionals in highly specialised fields and working in unique positions within leading international institutes, therefore potentially creating a specific viewpoint of HNL and its management. Despite the shared learning between multidisciplinary HCPs within the focus groups there was at times a hierarchy of power with clinical leads being present and potentially implicating hesitation in other professionals responding to questions before their superior [35, 36].

## Conclusion

The prevalence and chronicity of HNL demonstrates the fundamental role that self-management plays in the overall management of HNL after the completion of HNC treatment. HCPs involved in the delivery of care for patients with HNC require education and training on HNL to enable them to confidently provide timely and relevant patient information, comprehensively assess patients and make appropriate early referrals and support engagement with HNL self-management. Insights gained from the HCP perspective provide important elements to consider in the development of a dedicated HNL self-management intervention such as, mode of delivery, timing of information, terminology and clarification of roles, to not only encourage compliance of self-management techniques but to promote post-treatment HRQOL for patients with HNC.

## Supplementary Information

Below is the link to the electronic supplementary material.

Supplementary file1 (DOCX 20 KB)

## Data Availability

No datasets were generated or analysed during the current study.
