# Supplementary material for: Healthcare professionals understanding, perceptions and experiences of head and neck lymphoedema to promote self-management for patients following completion of head and neck cancer treatment: a qualitative study
Source: J Cancer Surviv. 2025 Jul 16;20(Suppl 1):810–22. doi: 10.1007/s11764-025-01868-4 (PMC13676533; doi:10.1007/s11764-025-01868-4)
Supplement: Supplementary file 1 [file 11764_2025_1868_MOESM1_ESM.docx]

Phase 1 - Topic guide for focus group - HCP

*Research title: Planning, developing and testing a tailored self-management lymphoedema intervention for patients who have completed treatment for head and neck cancer to improve health-related quality of life.*

Every effort will be made to ensure all participants are put at ease and the topic of head and neck lymphoedema following treatment will be introduced.

**HCPs perspectives and experience of head and neck lymphoedema challenges following treatment.**

**From your clinical experience:**

- Describe some of the challenges that patients and family members/carers encounter with head and neck lymphoedema(HNL) post-treatment?
- How do these challenges impact them? Probes: physically, emotionally, socially or in relationships?
- What is the reason for their challenges with lymphoedema post-treatment?
- Do the challenges and difficulties change over time?
- In everyday life, what impacts/concerns patients most when living with HNL? Probes: appearance or ability to perform everyday tasks? Other impacts or experiences with HNL?
- Interested in the sense of education. What are patients understanding of HNL?
- Best descriptors or terminology when providing education for HNL.
- Reflection on the most appropriate time point for this education.

**Facilitators/Barriers**

- What techniques or advice is provided to patients for HNL? (Probe: compression, MLD, SLD, skin care, exercises).
- Is there a particular technique that patients find most beneficial? If so, is there a reason? Does it vary?
- Does self-management have a role to play in the management of HNL? If so, explore how this could be implemented.
- What, if any, are the common reported facilitators and/or barriers in carrying out self-management for head and neck lymphoedema post-treatment?
- Any prompts to promote engagement/adherence to HNL techniques ?

**Support**

- What are some key areas that you believe patients need support with regarding their head and neck lymphoedema self-management at home?
- How can this support be provided to them (Probe: when and by who)?
- Does the family member have a role to play in HNL self-management?
- Is there any way to best support family members/carers?
- When is the best opportunity to provide support?
- What resources or methods would you use to support someone with head and neck lymphoedema at home post-treatment?
- Would you be confident in providing support for head and neck lymphoedema self-management challenges? (To patients, family members/carers).

**Intervention**

- We are interested in the education and understanding of patients and how best to provide this to them.
- It will be good to hear today from the experts what would be the most appropriate content of an educational resource? What information do you think should be provided?
- Timing of this information?
- Mode of sharing this information?
- Who is best placed to provide other patients with information on how best to manage post-treatment head and neck lymphoedema difficulties? (Probe: HCPs, other patients, relatives)?
- What is the best way for patients to access / get this information and support? (Probes: leaflet, online, workshop/health event, during follow-up clinic from HCPs)?
- What should it look like? The format? Leaflet? Online? Workshop/health event?
- Should it be for patients/family/HCPs?
- When would be a good time for you to hear about this support? Before/during/after treatment.
